# Vitamin D Deficiency and Its Predictors in a Country with Thirteen Months of Sunshine: The Case of School Children in Central Ethiopia

**DOI:** 10.1371/journal.pone.0120963

**Published:** 2015-03-30

**Authors:** Tolassa Wakayo, Tefera Belachew, Hassan Vatanparast, Susan J. Whiting

**Affiliations:** 1 Jimma University, Jimma, Ethiopia; 2 University of Saskatchewan, Saskatoon, Canada; Institute for Health & the Environment, UNITED STATES

## Abstract

Studies examining vitamin D status among children living in sunny climates indicated that children did not receive adequate vitamin D, however, this has not been looked at among children living in Ethiopia. In this study, we determined vitamin D deficiency and its predictors among school children aged 11–18 years, examining circulating 25-hydroxy vitamin D [25(OH)D]. The school-based cross-sectional study was conducted in schools in Adama Town (n = 89) and in rural Adama (n = 85) for a total sample of 174. Students were randomly selected using multi-stage stratified sampling method from both settings. Socioeconomic status of parents and demographic, anthropometric, sun exposure status and blood 25(OH)D levels were obtained. Vitamin D deficiency, defined as circulating levels of 25(OH)D <50 nmol/L, was found in 42% of the entire study participants. Prevalence of deficiency was significantly higher among students in urban setting compared to rural (61.8% *vs* 21.2%, respectively, *p*<0.001). After controlling for potential confounders using multivariable logistic regression model, duration of exposure to sunlight, amount of body part exposed to sunlight, place of residence, maternal education, body fatness, having TV/computer at home and socioeconomic status were significant predictors of vitamin D deficiency. The findings suggest that Vitamin D deficiency was prevalent in healthy school children living both in urban and rural areas of a country with abundant year round sunshine providing UVB, with the prevalence of deficiency being significantly higher among urban school children who were less exposed to sunlight. Behaviour change communication to enhance exposure to ultraviolet light is critical to prevent vitamin D deficiency in tropical country like Ethiopia. Further study is required to assess the deleterious effect of its deficiency on bone mineral homeostasis of growing children in Ethiopia during their most critical period of bone development.

## Introduction

Adolescence is the most critical period in skeletal development during which peak growth velocity is concomitant with an increase in bone mass. Thus, there is need for adequate vitamin D which is important for calcium and phosphate absorption as well as bone growth and accretion [1–5]. Vitamin D has other functions, such as modulation of immune function and regulation of cellular differentiation which are implicated in reducing risk of various diseases [2–6]. In humans, the main supply of vitamin D comes from its production in the skin following exposure to ultraviolet B radiation (UVB) at the wave length ranging from 280–315 nm. Naturally occurring dietary sources include: meat, fish, eggs, sun dried mushrooms [7–8]. Circulating 25-hydroxy cholecalciferol [25(OH)D] concentration is considered the best indicator of an individual’s vitamin D status, reflecting both cutaneous synthesis and dietary consumption of the nutrient [4,9,10]. Production of vitamin D_3_ in the skin depends on sunshine exposure, season, latitude, time of day, aging, skin covering clothes, the use of sun block, glass windows, and skin pigmentation [9,11–13].

There is a growing body of evidence indicating that vitamin D deficiency is a public health problem worldwide, affecting 30–80% of populations [14–17]. A high prevalence of vitamin D deficiency exists in tropical countries [10,18,19], particularly in children [20–24]. In Ethiopia, a recent study shows a problem in women [25]. However, there is no data in Ethiopia that documented the prevalence of vitamin D deficiency and its predictors among school children in Ethiopia.

Although Ethiopia is known as a country with 13 months of sunshine (12 months of 30 days each and 13^th^ month of 5 days, which will be 6 days every leap year), children attending schools, especially in urban areas, may be at increased risk for vitamin D deficiency because of the limited sun exposure, as they spend most of their time indoors every day. Thus, we undertook the present study in an urban and a rural setting to determine vitamin D status and its predictors among apparently healthy school children aged 11–18 years in urban of Adama Town and surrounding rural kebeles of Adama Woreda, Central Ethiopia. We hypothesized that there would be less vitamin D deficiency in rural subjects compared to urban subjects because of higher abundant sunshine exposure in rural setting due to different outdoor daily activities. We set out to identify predictors of vitamin D status among urban and rural school children, and examine effects of age, gender, sun exposure, body composition, and lifestyle factors on vitamin D status.

## Methods

### Subjects and design

**Fig 1:**
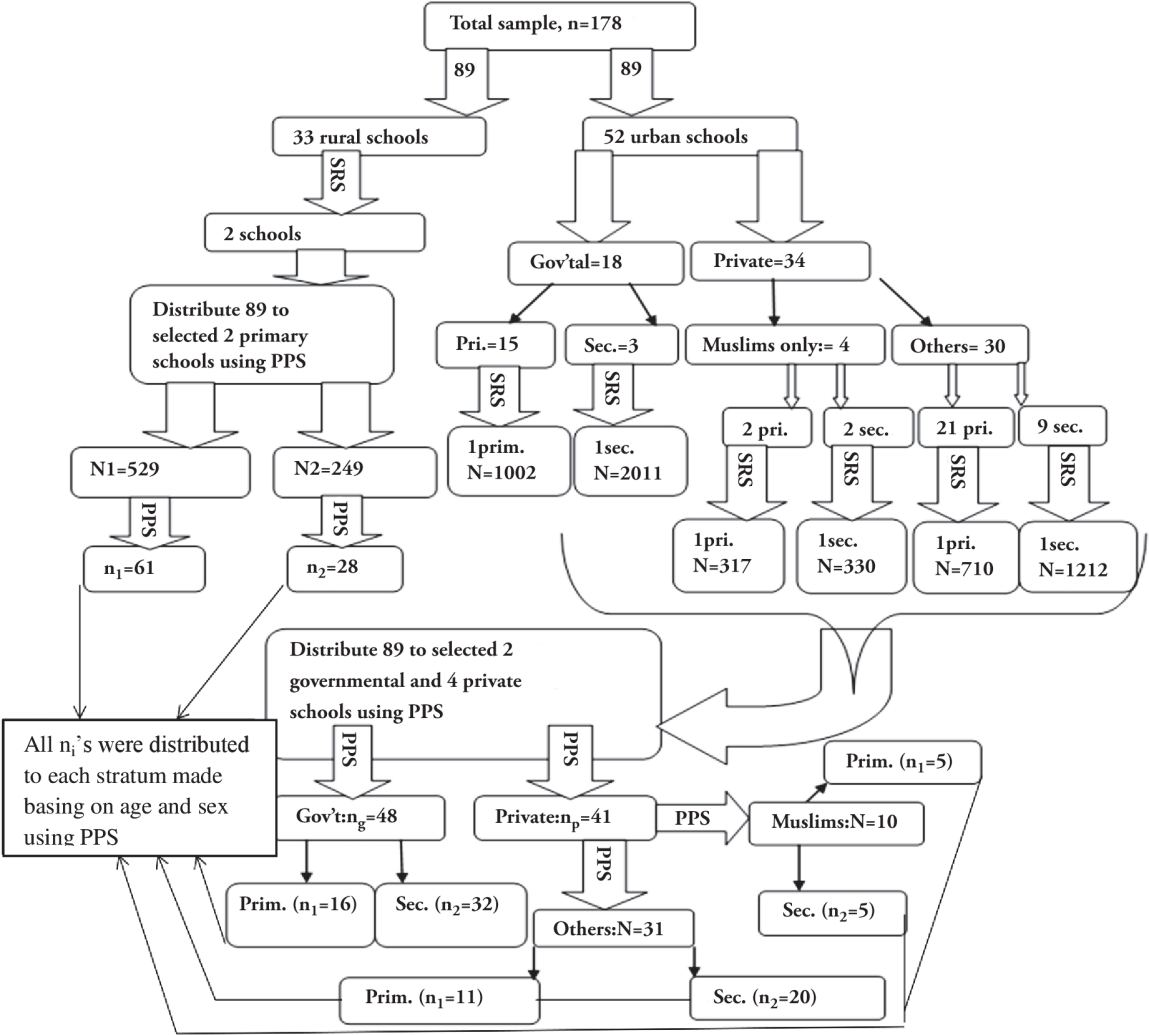
Flowchart of the sampling procedure of the institution-based cross-sectional study carried out to determine vitamin D deficiency and its predictors among school children in central Ethiopia. Abbreviations: SRS = Simple random sampling, PPS = Probability proportional to size allocation, Pri = Primary school, Sec = secondary school.

A school based cross-sectional study was conducted from May 20–June 22, 2013, in Adama City (n = 89) and in Rural Adama woreda (n = 85) located in Central Ethiopia (latitude: 8°33’- 8°36’N). To select study subjects from each study setting, multi-stage stratified random sampling procedure was used (**Fig. 1**). First, schools were selected randomly and sample sizes were allocated to each selected school using probability proportional to size allocation (PPS). Then, all children aged 11–18years in each selected school were stratified based on their age and gender using Microsoft Office Excel spreadsheet application and PPS was used again to distribute allocated samples in the first step to each school’s stratum. Finally, children from each stratum were selected using simple random sampling technique.

The sample size was calculated assuming an anticipated prevalence of vitamin D deficiency in rural subjects of 58% (24) and a 20% difference (increase) in urban subjects that was assumed in advance [26]. A sample size of 89 from each study setting was considered sufficient for results with 80% power and 95% confidence interval and an alpha error of 5%.

Children were excluded from the study using the following criteria: age outside the range of 11 year (minimum) or 18 year (maximum); reported medical history of diagnoses of liver or kidney diseases; a history of epilepsy; having skin damage such as major burn or dermatological problems.

### Ethics Statement

The complete study protocol was approved the by the Hawassa University Institutional Review Board (as the PI was a graduate student), by the University of Saskatchewan Research Review Board and National Research Ethical Review Committee of the Ethiopian Science and Technology Ministry. The parents/guardians of all the selected children were invited to visit the schools by the researcher and each school directors. The objectives of the study were fully explained to the parents/guardians and potential subjects in an open session, after which informed written consent was signed by the parents/guardians and then verbal assent was obtained from the children. A participation rate of 98% was achieved.

### Vitamin D Status

Each child had a finger prick by trained health workers, from which free-flowing blood drops were collected on blood spot cards as per ZRT laboratory instructions (www.zrtlab.com). At least two such usable (non-overlapping) drops were collected per subject. After air drying for at least 30 minutes, flaps were closed and placed in the sealed Ziploc bags with desiccant and moisture indicators, and taken to Oromia Public Health Research, Capacity Building and Quality Assurance Laboratory by principal investigator for storage at −80°C. Samples were then sent for analysis to ZRT laboratory (Oregon, USA), within 4 days. Circulating 25(OH)D was analyzed from dried blood spots, using a standard LC-MS/MS assay having intra-assay and inter-assay CVs of 8.1% to 9.2% and 12% to 13%, respectively. The ZRT Laboratory participates in DEQAS, and the blood spot is deemed equivalent to serum [27]. A cut-off for deficiency of <50 nmol/L was based on consideration of the Institute of Medicine’s use of it as an individual’s cut-off for vitamin D status based on bone health, and a recent endorsement of this value by European experts [28].

### Anthropometric Data

Body weight, height, and triceps skinfold thickness were measured using a precision digital scale (HD-318; TANITA), portable stadiometer, Holtain skinfold caliper, respectively. Children removed their shoes and jackets for height and weight measurements. Height, weight and skinfold thickness were measured 3 times and the mean was used for analysis. Body Mass Index (BMI) was calculated as weight in kilogram divided by height in squared meter (kg/m^2^). The triceps skinfold (TSF) was measured at the upper arm mid-point mark between acromion process of shoulder blade and olecranon process of ulna on the posterior surface of the left upper arm. Data for BMI and TSF were compared to WHO references for specific age and sex. Every morning and prior to each measurement, the weight scale was calibrated with a standard weight and instruments were calibrated according to the manufacturer’s recommendations.

### Sun exposure, body part exposed and skin color

Duration of exposure to sunlight and amount of body part exposed to sun were assessed using a pretested structured questionnaire developed from previous studies [29,30]. Questions asked about usual sun exposure duration and the amount of skin usually exposed for that specified duration. Skin color of participants was classified as “light brown”, “dark brown” or “very dark” as determined by observing untanned skin on the upper inner arm by the principal investigator.

### Socioeconomic Index

Socioeconomic index was developed as follows: first all study participants were asked about the ownership of fixed assets by their household with a score 1 given to those who own the asset and score of “0” given to those who did not own. Then all items asked were assessed for internal consistency and showed to be reliable with a cronbach’s alpha value of 0.82 (>0.7 is considered as reliable). Then principal component analysis was used to develop the wealth index. The first factors were taken and rank ordered into Tertiles.

### Statistical analysis

Data were entered in double, checked for missing values and outliers, and analyzed using SPSS for window (SPSS Inc. version 16.1, Chicago, Illinois). Mean values between groups were compared using independent sample t-test and one way analysis of variance after checking for normality and adjusting if necessary. When a significant difference was found, the multiple comparison test used was Tukey HSD post hoc test in ANOVA. Normality of the continuous variables was checked visually using Q-Q plots of residuals against the predicted values and using the Kolmogrov–Smirnov test. Logarithmic transformations were made as necessary. Correlations were evaluated using Pearson’s coefficients. The X^2^ tests were used to compare categorical data between groups. Circulating 25(OH)D concentrations were grouped into two (i.e. ≥50 nmol/L as “normal” and <50 nmole/L as “deficient”). To determine predictors of vitamin deficiency, we first carried out bivariate analyses to identify candidate variables for the multivariable model. Second, to identify the significant predictors of vitamin D deficiency, variables that had *p* <0.25 in the bivariate analyses were entered in the multivariable regression model. At this step, interaction between different variables was checked and collinearity diagnostics was done by checking the variance inflation factor and Pearson correlation coefficient. Variance inflation factor (VIF >3) and Pearson correlation coefficient (r >0.6 or < −0.6) were used to indicate the problem of multicollinearity among predictor variables themselves. After identifying predictor variables that had collinearity with specific predictor variable, they were excluded from the model and controlled for their potential confounding effect on specific predictor variable in multivariable logistic regression model using stepwise procedure. During each time, enter method of multivariable logistic regression model was used to identify the significant predictors at an Alpha level of 0.05. We also checked the interaction between each predictor variable and the study setting. No statistically significant interaction between study setting and other predictors was noted in our analyses. All tests were two-sided and *p* <0.05 was considered statistically significant. The results were reported as Odds Ratio and 95% CI.

## Results

A total of 174 students from urban (51.1%) and rural (48.9%) settings were enrolled in this study. Of these, 60.7% and 52.9%, respectively were females. The majority of urban (70.8%) participants were in the age group 15–18 year, while most (60%) of the rural students were aged 11–14 year. Few Muslim students were sampled in the rural setting, while in the urban setting there were close to equal numbers. Educational status of parents differed between urban and rural settings. The high proportion (84.7%) of households in the rural setting was farmers, while larger proportion of households in the urban setting was in the high socio-economic status based on wealth index (**Table 1).**

**Table 1:** Socioeconomic and demographic characteristics of schoolchildren in Central Ethiopia.

**Characteristics (n = 174)**		**Frequency (%)**	
		**Urban (n = 89)**	**Rural (n = 85)**
Gender	Male	35 (39.3)	40 (47.1)
	Female	54 (60.7)	45 (52.9)
Age groups	11–14	26 (29.2)	51 (60)
	15–18	63 (70.8)	34 (40)
Religion	Christians	55 (61.8)	84 (98.8)
	Muslims	34 (38.2)	1 (1.2)
Educational status (father)	No formal education	7 (7.9)	47 (55.3)
	Formal education	82 (92.1)	38 (44.7)
Educational status (mother)	No formal education	17 (19.1)	60 (70.6)
	Formal education	72 (80.9)	25 (29.4)
Occupation (Father)	Farmer	11 (12.4)	72 (84.7)
	Merchant	24 (27)	4 (4.7)
	Employed	54 (60.7)	9 (10.6)
Occupation (Mother)	House wife	52 (58.4)	78 (91.8)
	Merchant	17 (19.1)	1 (1.2)
	Employed	20 (22.5)	6 (7.1)
Socioeconomic index	Low	20(22.5)	22 (34.1)
	Medium	14 (15.7)	38 (44.7)
	High	55 (61.8)	18 (21.2)

Overall prevalence of vitamin D deficiency (serum 25(OH)D <50 nmol/L) was 42%. The proportion of deficiency was significantly higher among students in urban setting compared to those in rural setting (61.8% *vs* 21.2%, respectively: *p* <0.001). We also classified study participants using serum 25(OH)D of greater than >75 nmol/L and >50–74.9 nmol/L as sufficient and insufficient, respectively. Surprisingly, only few study participants, 3(3.4%) in urban and 12(14.1%) in rural were vitamin D sufficient while 31(34.8%) in urban and 55(64.7%) in rural were vitamin D insufficient. Mean serum 25(OH)D level were significantly lower for urban compared to rural students (*p* <0.001). Similarly, the mean serum 25(OH)D level among girls was significantly lower than that of boys (*p* <0.001). Additionally, statistically significant differences between the mean serum 25(OH)D levels of the following were found: younger higher than older; overweight lower than non-overweight based on classification of both BMI-for-age and TSF-for-age percentiles (Kappa = 0.709). However, there was no statistically significant difference between the mean serum 25(OH)D levels of respondents according to their skin color (*p* = 0.211). It was also observed that, the mean serum 25(OH)D levels of respondent groups varied significantly according to the duration of their exposure to sun light: weekly sun exposure on school days and weekend days, by amount of parts of the body exposed to the sun on school days and weekend days, and by socioeconomic status. For the socioeconomic status group, Tukey’s post-hoc tests revealed that these results were driven by the significantly lower serum 25(OH)D concentrations in the high socioeconomic group with respect to the other two groups: low socioeconomic and high socioeconomic (*p* <0.001), and middle socioeconomic and high socioeconomic (*p* <0.001). No significant pair wise difference in mean serum 25(OH)D were found between low and middle socioeconomic groups (*p* = 0.845) (**Table 2**).

**Table 2:** Circulating 25(OH)D levels according to study variables among schoolchildren in Central Ethiopia.

**Variable (n = 174)**	**Frequency**	**Serum 25(OH)D (nmol/L)** ^1^	***P* value**
All participants(n = 174)		54.5±15.9	
Study setting			
Urban	89	48.2±14.0	*p*<0.001
Rural	85	61.0±15.1	
Gender			
Male	75	60.3±16.9	*p*<0.001
Female	99	50.0±13.5	
Age groups			
11–14	97	57.3±14.1	p<0.001
15–18	77	52.2±16.8	
Religion			
Muslim	35	44.3±14.3	*p* <0.001
Christian	139	57.0±15.2	
BMI Classification			
≥85^th^ percentile	12	42.6±10.7	*p* = 0.007
<85^th^ percentile	162	55.4±15.8	
TSF Classification			
≥90^th^ percentile	18	44.0±10.2	*p* = 0.003
<90^th^ percentile	156	55.7±15.9	
Daily sun exposure on school days			
<30 min	33	40.7±10.5	
30–60 min	48	51.0±13.7	*p*<0.001
≥60 min	93	61.2±14.8	
Body part exposed to the sun on school days			
Face, hands & feet	46	41.7±11.0	*p*<0.001
More than face, hands, & feet*	126	59.1±14.8	
Daily sun exposure on weekend days			
<30 min	43	42.5±12.4	*p*<0.001
30–60 min	32	52.0±11.9	
≥60 min	99	60.5±15.2	
Body part exposed to the sun on weekend days			
Face, hands & feet	31	41.0±11.8	*p*<0.001
More than face, hands, & feet*	143	57.4±15.1	
Skin color			
Light brown	39	54.0±11.6	
Dark brown	98	56.1±17.0	*p* = 0.211
Very dark	37	50.7±16.4	
Socioeconomic index			
Low	49	65.2±12.3	
Middle	52	64.1±11.4	*p*<0.001
High	73	40.0±8.0	

1Values are mean ± SD

*Additional exposure at the neck, forearms, upper arms, or legs.

Abbreviations used: 25(OH)D, 25-hydroxyvitamin D; SD, standard deviation; BMI = body mass index;

TSF = triceps skin fold thickness

A subgroup analysis was done on girls by religion. Muslim girls (n = 28) had significantly lower serum 25(OH)D levels (40.8±12.6 nmol/L) than Christian girls (53.7±12.1 nmol/L). There was a trend (*p* = 0.06) toward an association between religion of the girls and their vitamin D status from chi-square test (crude OR = 2.53[0.91, 7.02]). For Muslim girls, we attempted to test those who wore Hijab regularly as school uniform (n = 6) and those Muslim students who did not wear Hijab regularly (n = 22), but who also wore clothing covering forearms during school time. Mean serum 25(OH)D concentration of Hijab-wearing girls (27.3±13 nmol/L) was significantly lower than those who were not wearing (43.9±9.2 nmol/L). However, in the chi-square test there was no significant association between dressing style and vitamin D status of Muslim girls.

The significant predictors of vitamin D status in the present study after controlling for potential confounders using logistic regression model were study setting, maternal education, body fatness, having television/computer in the home, socioeconomic status, duration of sun exposure on school days, amount of body parts exposed to the sun on school days, duration of sun exposure on weekend days and amount of body parts exposed to the sun on weekend days **(Tables 3 and 4)**.

**Table 3:** Sociodemographic and anthropometric predictors of vitamin D status in logistic regression analysis for Ethiopian schoolchildren^a^.

**Variables (n = 174)**	**Vitamin D status**		**COR (95%CI)**	**AOR (95%CI)**
	**Deficient**	**Normal**		
	**Number (%)**	**Number (%)**		
Study setting^1^				
Rural	18 (21.2%)	67 (78.8%)	Reference	Reference
Urban	55 (61.8%)	34 (38.2%)	6.02 (3.07, 11.81)	10.53 (3.94, 28.17)
Gender				
Male	22 (29.3%)	53 (70.7%)	Reference	Reference
Female	51 (51.5%)	48(48.5%)	2.56(1.34, 4.83)	1.76 (0.81, 3.83)
Age groups				
11–14	25 (32.5%)	52 (67.5%)	Reference	Reference
15–18	48 (49.5%)	49 (50.5%)	2.04 (1.1, 3.79)	1.43 (0.66, 3.09)
Religion				
Christians	49(35.3%)	90(64.7%)	Reference	Reference
Muslims	24(68.6%)	11 (31.4%)	4.01 (1.81, 8.87)	1.61 (0.6, 4.32)
Education (Father)				
Formal education	58 (48.3%)	62 (51.7%)	2.43 (1.21, 4.87)	2.4 (0.96, 5.98)
No formal education	15 (27.8%)	39 (72.2%)	Reference	Reference
Education (Mother)^2^				
No formal education	22 (28.6%)	55 (71.4%)	Reference	Reference
Formal education	51 (52.6%)	46 (47.4%)	2.77 (1.47, 5.23)	2.74 (1.23, 6.12)
BMI Classification				
<85^th^ percentile	64 (39.5%)	98 (60.5)	Reference	Reference
≥85^th^ percentile	9 (75%)	3 (25%)	4.59 (1.2, 17.62)	4.67 (0.7, 31.07)
TSF Classification^3^				
<90th percentile	97 (62.2%)	59 (37.8%)	Reference	Reference
≥90th percentile	14 (77.8%)	4 (22.2%)	5.96(1.81, 18.31)	6.1 (1.24, 28.57)
Have TV/PC at home^4^				
No	19 (23.2%)	63 (76.8%)	Reference	Reference
Yes	54 (58.7%)	38 (41.3%)	4.71 (2.44, 9.12)	7.84 (3.19, 19.27)
Socioeconomic index^5^				
Low	16 (27.6%)	42 (72.4%)	Reference	Reference
Medium	17 (32.1%)	36 (67.9%)	1.3 (0.58, 2.93)	1.72 (0.59, 5.03)
High	40 (65.6%)	21 (34.4%)	5.24 (2.4, 11.42)	9.4 (3.19, 27.51)

^*a*^ ***Predictors in Table 3 and Table 4 were used simultaneously in the same multivariable logistic regression model.***

*PC = Personal computer*, *TV = Television*, *BMI = Body mass index*, *TSF = Triceps skinfold thickness*. *COR = Crude Odds Ratio; AOR = Adjusted Odds Ratio; CI; confidence interval; BMI = body mass index; TSF = triceps skin fold thickness confidence interval*.

Definition (cut-off point) for vitamin D deficiency = serum 25(OH)D < 50 nmol/L.

*1 Adjusted for age group*, *religion*, *parental education*, *sun exposure*, *TV/computer and socioeconomic index*.

*2 Adjusted for paternal education*, *sun exposure*, *setting and socioeconomic index*.

*3 Adjusted for BMI*, *sun exposure*, *setting and socioeconomic index*.

*4 Adjusted for setting*, *parental education*, *sun exposure*, *and socioeconomic index*.

*5 Adjusted for setting*, *parental education*, *sun exposure and TV/computer*.

**Table 4:** Predictors of vitamin D status related to sunlight exposure in logistic regression analysis for Ethiopian schoolchildren^a^.

**Variables (n = 174)**	**Vitamin D status**		**COR (95%CI)**	**AOR (95%CI)**
	**Deficient**	**Normal**		
	**Number (%)**	**Number (%)**		
Duration of daily sun exposure on school days^6^				
≥60 min	21 (22.6%)	72 (77.4%)	Reference	Reference
30–60 min	25 (52.1%)	23 (47.9%)	3.79 (1.77, 7.86)	5.58 (2.25, 13.85)
<30 min	27 (81.8%)	6 (18.2%)	15.43 (5.62,)	13.92 (4.3, 45.1)
Amount of body parts exposed to the sun on school days^7^				
More than* face, hands and feet	37 (28.9%)	91(71.1%)	Reference	Reference
Face, hands and feet	36 (78.3%)	10 (21.7%)	8.85 (3.99, 19.67)	13.38 (4.69, 38.21)
Duration of daily sun exposure on weekend days^8^				
≥60 min	24 (24.2%)	75 (75.8%)	Reference	Reference
30–60 min	18 (56.2%)	14 (43.8%)	4.02 (1.74, 9.27)	9.41 (3.35, 26.39)
<30 min	31 (72.1%)	12 (27.9%)	8.07 (3.59, 18.14)	7.25 (2.53, 20.75)
Amount of body parts exposed to the sun on weekend days^9^				
More than face, hands and feet*	48 (33.6%)	95 (66.4%)	Reference	Reference
Face, hands and feet	25 (80.6%)	6 (19.4%)	8.25 (3.17, 1.46)	19.57 (5.53, 9.21)
Skin color				
Light brown	14 (35.9%)	25 (64.1%)	Reference	Reference
Dark brown	39 (39.8%)	59 (60.2%)	1.18 (0.55, 2.55)	1.18 (0.46, 3.13)
Very dark	20 (54.1%)	17 (45.9%)	2.1 (0.84, 5.27)	1.26 (0.39, 4.1)

^*a*^ ***Predictors in Table 3 and Table 4 were used simultaneously in the same multivariable logistic regression model.***

* Additional exposure at the neck, forearms, upper arms, or legs.

Abbreviations used: COR = Crude Odds Ratio; AOR = Adjusted Odds Ratio; CI; confidence interval; BMI = body mass index; TSF = triceps skin fold thickness confidence interval.

Definition (cut-off point) for vitamin D deficiency = serum 25(OH)D < 50 nmol/L.

*6 Adjusted for setting*, *sun exposure on weekend days and socioeconomic index*.

*7 Adjusted for part of body exposed to the sun on school days*, *sun exposure*, *setting*, *and socioeconomic index*.

*8 Adjusted for setting*, *sun exposure on school days and socioeconomic index*.

*9Adjusted for part of body exposed to the sun on weekend days*, *sun exposure*, *setting*, *and socioeconomic index*.

Results of multivariable logistic regression analyses showed that students living in urban setting had 10.53 times more odds of being vitamin D deficient compared to those living in rural setting (AOR = 10.53[3.94, 28.17). Having a higher body fat (TSF ≥90^th^ percentile) was associated with 6 times more odds of being vitamin D deficient compared to having less body fat (TSF <90^th^ percentile) (AOR = 6[1.24, 28.57]). The odds of having vitamin D deficiency among students whose mothers had formal education was 2.74 times higher compared to students whose mothers had no formal education (AOR = 2.74[1.23, 6.12]). Duration of daily exposure to sun light and amount of body parts exposed to the sun on each school day and each weekend day were found to be significant predictors of vitamin D deficiency, but skin color was not associated. Students from high socio-economic status had 9.4 times more odds of being vitamin D deficient compared to students from low socio-economic status. Similarly, having TV/computer at home increases the likelihood of vitamin D deficiency by 7.84 times.

## Discussion

The present study showed that prevalence of vitamin D deficiency (serum 25(OH)D <50 nmol/L) was 42% in all school children, with students in urban setting being more likely to be deficient than their rural counterparts. Serum 25(OH)D levels <50 nmol/L were seen in 61.8% and 21.2% of urban and rural children, respectively (*p* <0.001). Our finding for a urban-rural difference is in keeping with studies conducted in other developing countries [26, 31–36], with the general assumption that rural populations are outdoor workers. An adequate, even optimal level of 25(OH)D has been reported in Africans living a traditional herder lifestyle [37].

Dietary vitamin D_3_ is found in animal-based foods such as meat, eggs, and fish [8]. Vitamin D_2_ is made upon sun exposure of certain fungi. We detected 5 students (3 from rural and 2 from urban settings) who had serum 25(OH)D >10 nmol/L in the form of vitamin D_2_ suggesting a significant dietary contribution to vitamin D level from fungi. A common food eaten by most subjects was yeast fermented Injera. It may be the method of production of of Injera or other cereal based fermented foods that might have contributed to significant D_2_ levels. The frequency of consumption of animal source foods potentially containing vitamin D_3_ was once weekly in the urban setting and mostly null in rural areas. Therefore, it is possible to conclude that our rural study subjects were getting their vitamin D from exposure to sun light, while urban subjects may have some dietary source. In some countries however, rural children may have indoor work and may wear concealing clothes. Harinarayan *et al*. (2008) found no association between location (urban and rural) and 25(OH)D levels in both boys and girls [33].

In the Middle East, many studies conducted in urban setting [38–42] found very high prevalence of vitamin D deficiency in contrast to Ethiopian children from the same setting. A possible explanation for this could be a very limited time spent outdoors due to the extreme climate and more commonly practiced wearing of concealing clothing in Middle East countries [15]. Studies in urban India [26,33,43,44] also showed higher prevalence of vitamin D deficiency than our urban finding, possibly due to the fact that most of Indian children may have indoor work and may wear concealing clothes.

The present study also showed that overweight/obese students (having TSF ≥90^th^ percentile) were more vitamin D deficient compared to those who were non-overweight/obese (having TSF <90^th^ percentile). Similar findings were reported from studies [36,45–46] where overweight/obese children were found to have lower serum 25(OH)D than non overweight/obese children. Wortsman *et al*. (2000) reported that vitamin D deficiency may be due to decreased bioavailability of vitamin D_3_ from cutaneous and dietary sources because of its deposition in body fat compartments [47]. However, our data suggest that body fatness is higher in subjects from better SES families, and we cannot eliminate the possibility of lifestyle (indoor activities).

Webb and Engelsen (2006) reported that a person exposing hands, face and arms for the time it takes to generate one minimal erythemal dose (MED) would make 1000 IU vitamin D_3_. Time to achieve an MED varied by skin color [48]. These factors could explain the protective effect that we observed in students who had more sun exposure time and more body parts exposed to the sun against vitamin D deficiency, our study subjects having skin types IV, V, and VI. Although our findings showed that Muslim girls who wore Hijab as school uniform regularly had significantly low mean serum 25(OHD), the result was inconclusive since the number of girls who wore Hijab regularly was very small and thus, might compromise the power to detect a truly existing difference of vitamin D status between the two groups. Students from high socio-economic status category were more likely to develop vitamin D deficiency compared to those whose families were in the low socio-economic status. Few studies have examined this factor for school children. Maddah *et al*. (2009) reported a similar findings in that women from lower socioeconomic background had higher serum 25(OH)D compared to their counterparts from higher socioeconomic background [35]. One reason may be less time spent in the sun, which may be interpreted as having more indoor activities (e.g., having computer, TV) and/or having fewer outdoor chores which would be related to farming households. An additional factor may be higher energy intakes of those in high vs. low socio-economic status leading to being overweight/obese [44]. We also observed that overweight/obese children had poorer vitamin D status which is consistent with reports of other studies [36,45,46].

Finally, educational status of mothers of children enrolled under this study had a statistically significant association with their children vitamin D status, in which, children whose mothers had formal education were found to be more likely to develop vitamin D deficiency as compared to those whose mothers do not had formal education after adjusting for study setting. In contrast, researchers from the USA [49,50] and Middle East [15] reported no association between maternal education and their children vitamin D status. For our results, there are two possible scenarios. First, more educated mothers may tend to avoid direct sun exposure through staying indoors or using sun screens, for themselves and their children as much as possible, due to skin cancer risk or merely to avoid sun burns or excessive tanning [35]. This is further supported by the fact that significantly higher and lower percentages (28.9% *vs* 6.5% and 33% *vs* 79.2%) of our study subjects whose mothers had formal education had duration of exposure to sunlight of <30 minutes and >60 minutes, respectively. Alternatively, more women with higher education were living in the urban setting which could explain the observed difference.

Vitamin D is an essential nutrient for linear growth of bones and for reaching peak bone mass among children and adolescents. The government of Ethiopia has targeted children and adolescents in the national nutrition program for accelerated stunting reduction and various interventions are underway. The high prevalence of vitamin D deficiency demonstrated by this study in a country where there is ample sunlight throughout the year (13 months of sunshine) calls for arguments to include behaviour change communications on the importance of exposure to sunlight. This could be done through inclusion of key messages in the school curricula in the long term and through establishing school nutrition clubs and other relevant educational strategies in the short run to curb the long term complications of vitamin D deficiency.

In this study, we acknowledge limitations. We used cross-sectional study exploring the association between vitamin D status and its predictors and thus a causal association between the two factors cannot be established. Although level of exposure to sunlight varies by season, this influence on serum 25-hydroxyvitamin D levels was not checked for the same reason of cross-sectional nature of the study design that we employed. We did not consider the design effect for the multi-stage sampling procedure that we employed as the cost of laboratory analyses was high. Hence, the study was based on small sample size that may not reflect the association for predictors that were not significantly associated with vitamin D status. We did not measure actual sun exposure and body parts exposed to the sun in our study and relied on self-reported data. Skin color was hard to measure and we were uncertain if the three distinctions we used were physiologically relevant. However, this study has some strength. The study included study subjects from two settings (urban and rural) so that it could provide insight into the vitamin D status of urban and rural school children in Ethiopia.

In conclusion, the present study demonstrated that vitamin D deficiency was prevalent among healthy school children in both urban and rural settings, with the prevalence being significantly higher among urban school children, which is unacceptable phenomenon in a country where there is ample sunshine throughout the year free of charge. Study setting, maternal education, TSF, duration of sun exposure, amount of body parts exposed to the sun, having TV/computer in the home and socioeconomic status were significantly associated with vitamin D status of our study subjects. With an increasing urbanization in the wake of globalization, countries such as Ethiopia need to prepare for increasing vitamin D deficiency. Further study is required to assess the deleterious effect of its deficiency on bone mineral homeostasis of growing children in Ethiopia during their most critical period of bone development. Very importantly, behaviour change communication to enhance exposure to ultraviolet light is critical to prevent vitamin D deficiency in tropical country like Ethiopia.

## Supporting Information

S1 Data (SAV)
